# Editorial

**DOI:** 10.1093/europace/euac230

**Published:** 2022-12-27

**Authors:** Angelo Auricchio

**Affiliations:** Division of Cardiology, Istituto Cardiocentro Ticino - EOC, Via Tesserete 48, 6900 Lugano, Switzerland


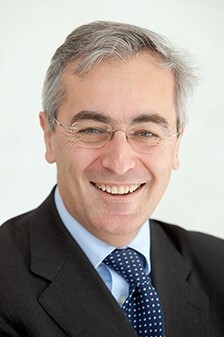


As recently announced, on 1 January 2023, I have the great honour to take over the position of Editor-in-Chief of the *EP Europace* journal and succeeding Prof. Gerhard Hindricks. I would like to take this opportunity to thank Prof. Hindricks and his whole Editorial Board for handing over a very successful journal dedicated to a fast-expanding field in cardiovascular medicine, cardiac electrophysiology. The hard work, dedication, and enthusiasm of Prof. Hindricks and of all editorial board members have made *EP Europace* one of the most appreciated journals in the growing family of journals dedicated to cardiac arrhythmia disorders diagnosis and management. With an impact factor of 5.486 (year 2022), *EP Europace* is among the top 4th sub-specialty journals.

With this editorial, I would like to briefly review the most significant achievements of *EP Europace* during the current year and to share with you some personal strategic thoughts about journal development in the hope of best matching the needs and expectations of our readership in the future.

Since the first journal issue in 1999 under the leadership of its founding editor Prof. Richard Sutton,^1^ and then Prof. John Camm’s and Prof Hindricks’ management, *EP Europace* has experienced a steady growth in high-quality manuscripts submitted from all around the world. The more than 1200 annually submitted articles, a good proportion of them being published as open access, accompanied by a huge increase in the number of downloaded articles confirm the recognition of the scientific and education value of the journal. Along with the increase in the number of yearly submissions, the manuscript acceptance rate has progressively reduced to about 17%, which ensures the publication of high-quality, clinically, and scientifically impactful manuscripts.

Being the official journal of the European Heart Rhythm Association (EHRA), *EP Europace* represents the main communication platform for our Association. Indeed, in 2022, the journal has published a total of 19 EHRA scientific documents and EHRA-related scientific initiatives such as EHRA surveys. EHRA scientific documents developed by EHRA Scientific Document Committee alone or in collaboration with other internationally recognized scientific organization are a very important source of current knowledge in the field; they provide state-of-the-art treatment standards and recommendations. Furthermore, they place upcoming technologies and novel research avenues in the proper scientific framework. An excellent and most recent example of clinical practice-oriented scientific document is represented by the EHRA practical guide on how to use digital devices to detect and manage arrhythmias^2^ with an Altmetric score of 235 by 1 December 2022. Another good example of EHRA-initiated international scientific document is represented by the expert consensus statement on the state of genetic testing for cardiac diseases issued by EHRA in collaboration with Heart Rhythm Society, Asia Pacific Heart Rhythm Society, and Latin Americas Heart Rhythm Society^3^ with an Altmetric score of 220 by 1 December 2022. In addition, *EP Europace* regularly publishes important scientific contributions by the EHRA Scientific Initiative Committee, the EHRA Surveys. These reports collect clinically relevant data on recent trends in the different areas of cardiac arrhythmia management including non-invasive diagnostic tools, use of cardiac imaging technologies, implantable cardiac devices, techniques for cardiac ablation, and patient’s follow-up technologies. *EP Europace* is also affiliated with the Working Groups on e-Cardiology and Cardiac Cellular Electrophysiology of the European Society of Cardiology and enjoys regular scientific contributions by these two working groups.

In short, *EP Europace* represents a leading journal in the field of cardiac arrhythmias and provides a good balance between state-of-the art reviews, scientific documents, and original scientific contributions. In approaching its 25-year anniversary, the question is: where to go from here?

In my role as Editor-in-Chief, I feel very privileged to work with five outstanding scientists who will act as Executive Editors: Katja Odening (Bern, Switzerland), Elijah Behr (London, UK), Giuseppe Boriani (Modena, Italy), Luigi Di Biase (New York, USA), and Jens Cosedis-Nielsen (Aahrus, Denmark), and a large group of international key-opinion leaders as Deputy and Associate Editors. They will support the journal activities on a daily basis, assisting to identify new strategic editorial opportunities and help to safely navigate in challenging waters.

The raising demand to access breaking news or ‘just happening’ news in our society, supported by the massive use of social media, is contagious to any field of human knowledge including that of arrhythmia management and cardiac electrophysiology. Such society requirements—or expectations—translate into science in a rapid adaptation of the publication model. It is the daily experience of every journal editorial board that scientists request shorter review time of their research findings, expect high-quality feedback and comments by their peers, and demand rapid access to the international audience of their highly valuable accepted scientific content throughout multiple communication channels. To best match authors’ expectations, journal editorial boards and publishers including the new Editorial Board of *EP Europace* and its publisher, Oxford University Press (OUP), are steadily adapting the publication workflow and undertaking editorial measures to cope with it. The newly appointed Editorial Board of *EP Europace* journal has re-designed the manuscript handling process and is committed to meaningfully reducing the turnaround time of each submitted manuscript while ensuring a fair and balanced peer-review process. Furthermore, to ensure a nearly immediate sharing with the scientific community of the original content of an accepted manuscript, starting on 1 April 2023, the author’s accepted manuscript will be published as a PDF online within a couple of days from when the author signs their publishing licence. A digital object identifier will be given to each manuscript which will allow immediate consultation of the work, article citation, and eventually a significant chance and opportunity for the transmission of messages and article-related data within connected communities. Together with EHRA, the European Society of Cardiology (ESC) journal family, and other scientific partners, the entire Editorial Board, and I sincerely hope that each of our readers will take the opportunity to increase the visibility of results published in *EP Europace* journal.

To further expand the international visibility of each accepted article including developing countries or geographies with limited access to science, *EP Europace* will be the first journal in the sub-specialty field of cardiac arrhythmias management to become a full Open Access journal.

From January 2023, all papers published in *EP Europace* will be freely available to all readers. This will mean the research in the Journal will have a wider reach and can therefore be expected to have a wider impact. Open Access charges apply to authors (the latest charges can be found here: https://academic.oup.com/europace/pages/General_Instructions#Open%20access%20options%20for%20authors) and—very importantly—there are discounts available to EHRA members. As with all fully Open Access journals published by OUP, there is no charge for papers where a corresponding author submits from a country listed in OUP’s developing countries initiatives (https://academic.oup.com/pages/purchasing/developing-countries-initiative/participating-countries-and-regions). Additionally, OUP has a large number of ‘Read & Publish’ agreements in place, which means many authors will have their open access fees covered by their institution. To learn if your institution has a Read & Publish agreement in place and to find out how to check whether your paper can be covered by the funding available, please see https://academic.oup.com/pages/open-research/read-and-publish-agreements/participating-journals-and-institutions.

There is no doubt that the diagnosis and treatment of both atrial fibrillation and ventricular arrhythmias still represent huge clinical unmet needs, and thus the scientific and education focus of the journal will continue to be on these cardiac disorders. Moreover, implantable cardiac device therapies for rhythm disorders will continue to play another important role in the journal. However, the growing and expanding role of cardiac imaging and cardiac genetics in improving and personalizing the approach to arrhythmia management, appropriate device selection, and sudden cardiac death prevention well beyond the traditional ‘ejection fraction of the left ventricle’ will also find major attention in the journal. Indeed, a large and expanded group of Deputy and Associate Editors, all key opinion leaders in the field of cardiac genetics, marks the new journal focus. Therefore, we encourage our readers to consider *EP Europace* as one of the scientific discussion and publication forums for their novel and exciting research findings. The newly appointed editorial board of *EP Europace* journal also recognizes the significant role played by basic research for the progress of our field; thus, we will particularly welcome scientific contributions with a strong translational message including the area of computational modelling and simulation. Other very relevant scientific and educational areas are represented by the modern approach to syncope, the management of paediatric arrhythmic disorders, and last but not least, electrocardiology, which represents the ‘alpha-and-omega’ in clinical electrophysiology.

From 1 January 2023, there will be a major change in article categories and manuscripts requirements. Several additional manuscripts categories will be now considered, being ‘Controversy’, ‘Research letter’, ‘Trial design’, and the newest ‘Practical EP’. The new Editorial Board and I would be very happy to receive manuscripts of high research and clinical scientific significance, thus fulfilling the requirements of a ‘Fast Track’ article; we commit to a very short manuscript handling and publication time. Moreover, the reference list accompanying original clinical and translational research manuscripts has been significantly enlarged up to 50 references, and there is no limitation to the number of allowed figures and tables. I encourage authors to view the section ‘Instruction to Authors’ (https://academic.oup.com/europace/pages/General_Instructions), which details the new manuscript categories and article requirements. A major novelty is also represented by the cancellation of acceptance of case report. Therefore, we encourage our readers to consider *European Heart Journal – Case Report*s for the submission of highly educational, valuable case reports, images, and quality improvement projects.

I am very much looking forward to a productive, engaging, and enjoyable editorial time conducive to high-quality scientific and educational journal content. This is also the right time to express heartfelt thanks to all members of the new *EP Europace* Editorial Board for accepting my invitation to spend time in advising me as Editor-in-Chief and in performing invaluable editorial activities, the entire OUP editorial team for their unrestricted commitment to the new journal course, to my colleagues at Istituto Cardiocentro Ticino for their extraordinary support and encouragement, and all of you who in the past have continuously sustained the journal as author, editor, reviewer, or readers and will continue to do so in the future!

## References

[1] Sutton R . The first European journal on cardiac electrophysiology and pacing, the European Journal of Cardiac Pacing and Electrophysiology. Europace 2011;13:1663–4. 22089974 10.1093/europace/eur358

[2] Svennberg E, Tjong F, Goette A, Akoum N, Di Biase L, Bordachar P *et al*. How to use digital devices to detect and manage arrhythmias: an EHRA practical guide. Europace 2022;6:979–1005. 10.1093/europace/euac038 PMC11636571 35368065

[3] Wilde AAM, Semsarian C, Márquez MF, Shamloo AS, Ackerman MJ, Ashley EA *et al*. Developed in partnership with and endorsed by the European Heart Rhythm Association (EHRA), a branch of the European Society of Cardiology (ESC), the Heart Rhythm Society (HRS), the Asia Pacific Heart Rhythm Society (APHRS), the Latin American Heart Rhythm Society (LAHRS). EHRA/HRS/APHRS/LAHRS expert consensus statement on the state of genetic testing for cardiac diseases. Europace 2022;8:1307–67. 10.1093/europace/euac030 PMC9435643 35373836

